# Detección de *Toxoplasma gondii* en agua para el consumo humano proveniente de jagüeyes del área rural del municipio de Sincelejo

**DOI:** 10.7705/biomedica.5858

**Published:** 2021-05-31

**Authors:** Diana Marcela Campo-Portacio, Luisa Fernanda Guerrero-Velásquez, Angie Patricia Castillo-García, Kelly Orozco-Méndez, Pedro José Blanco-Tuirán

**Affiliations:** 1 Investigaciones Biomédicas, Universidad de Sucre, Sincelejo, Colombia Universidad de Sucre Investigaciones Biomédicas Universidad de Sucre Sincelejo Colombia; 2 Programa de Maestría en Biología, Universidad de Sucre, Sincelejo, Colombia Universidad de Sucre Universidad de Sucre Sincelejo Colombia

**Keywords:** Toxoplasma, agua cruda, reacción en cadena de la polimerasa, determinantes sociales de la salud, Toxoplasma, raw water, polymerase chain reaction, social determinants of health

## Abstract

**Introducción.:**

La toxoplasmosis es una zoonosis que se transmite por vía oral al consumir alimentos contaminados con cualquier forma infectiva de *Toxoplasma gondii.* Su transmisión por agua ha sido documentada en varios países, incluido Colombia. Al no disponer de agua potable, la población rural de Sincelejo podría estar en riesgo de contraer toxoplasmosis por esta vía.

**Objetivo.:**

Evaluar la contaminación por *T. gondii* del agua para consumo humano proveniente de jagüeyes de la zona rural de Sincelejo y establecer su relación con diferentes determinantes sociales de la salud en el área de estudio.

**Materiales y métodos.:**

Mediante PCR anidada, se evaluaron 96 muestras de agua obtenidas en 48 fincas ubicadas en ocho corregimientos rurales de Sincelejo. En cada finca se obtuvieron dos muestras: una de agua cruda de jagüey y otra destinada al consumo directo. En cada finca se hizo una encuesta para recolectar información sobre características físicas de la vivienda, presencia de gatos, y disponibilidad de agua y sus usos. Las relaciones estadísticas se evaluaron con el test de Fisher.

**Resultados.:**

De las 96 muestras analizadas, 13 resultaron contaminadas con *T. gondii* (13,5 %): nueve de agua cruda y cuatro de agua para el consumo directo. No se encontró asociación estadística entre las muestras positivas y los determinantes sociales de la salud evaluados (p>0,05).

**Conclusión.:**

La población rural de Sincelejo podría estar en riesgo de contraer toxoplasmosis por el uso y consumo del agua proveniente de sus jagüeyes. La contaminación de estos cuerpos de agua por *T. gondii* puede estar influenciada por otros determinantes sociales de la salud no contemplados aquí.

## Abstract (en)

**Introduction:**

Toxoplasmosis is an orally-transmitted zoonosis that may appear after consuming food contaminated with any infective form of *Toxoplasma gondii.* Its transmission by water has been reported in several countries including Colombia. The rural population of Sincelejo could be at risk of contracting toxoplasmosis through this route given that they lack potable water.

**Objective:**

To evaluate *T. gondii* contamination in water for human consumption from water wells *(jagüeyes)* in the rural area of Sincelejo and establish its relationship with different social determinants of health in the study area.

**Materials and methods:**

Using nested PCR we evaluated 96 water samples obtained from 48 farms located in eight rural townships in Sincelejo. We took two samples in each farm: one of raw water from water wells and the other intended for direct consumption. We conducted a survey on each farm to collect information on the physical characteristics of dwellings, the presence of cats, and the availability and uses of water. Statistical relationships were evaluated through Fisher tests.

**Results:**

Of the 96 samples analyzed, 13 were contaminated with *T. gondii* (13.5%): Nine corresponded to raw water and four to water for direct consumption. No statistical association was found between the positive samples and the social determinants of health under evaluation (p>0.05).

**Conclusion:**

The rural population of Sincelejo could be at risk of contracting toxoplasmosis through the use and/or consumption of water from its water wells. The contamination of these water bodies by *T. gondii* may be influenced by unstudied social determinants.

La toxoplasmosis es una zoonosis que afecta a la población mundial. La evidencia sugiere que un tercio de la población está infectada, razón por la cual se considera un problema de salud pública con gran impacto socioeconómico en términos de morbilidad y costos por el cuidado de los enfermos ^1^.

La infección suele ser asintomática en la mayoría de los pacientes inmunocompetentes y, de llegar a desarrollar síntomas, estos son leves, inespecíficos y de resolución espontánea. Los más frecuentes incluyen dolor muscular, astenia, cefalea e inflamación de los ganglios linfáticos. La toxoplasmosis cobra mayor importancia clínica cuando es congénita o hay reactivación de la infección en pacientes inmunocomprometidos ^2^.

Su agente causal es el protozoario parásito *Toxoplasma gondii,* el cual infecta al hombre y a la mayoría de animales homeotermos. Los huéspedes definitivos de *T. gondii* son los felinos, pues son los únicos animales que pueden eliminar ooquistes, forma infectiva del parásito, en las heces y diseminar así la infección en el medio ambiente ^3^. Los humanos pueden adquirir el parásito por la ingestión de carne mal cocida infectada con quistes tisulares de *T. gondii* (con bradizoítos), por la ingestión de ooquistes (con esporozoítos) presentes en frutas y verduras sin lavar y agua sin tratar, o por transmisión vertical ^4^.

En Colombia, la vigilancia epidemiológica de la enfermedad está enfocada principalmente a la prevención de las manifestaciones clínicas importantes: toxoplasmosis congénita y reactivación en inmunocomprometidos. Sin embargo, ambas formas clínicas siguen siendo un problema de salud importante para el país. Cada año aparecen entre 2 y 10 casos de infección congénita por cada 1.000 nacidos vivos ^5^. Además, la toxoplasmosis cerebral en pacientes infectados con HIV sigue siendo un problema importante de salud ^6^^-^^8^.

Los estudios nacionales para determinar las principales vías por las cuales la población colombiana adquiere el parásito, son escasos. Se han hecho algunos para detectar el ADN del parásito en carnes de consumo humano en diferentes ciudades ^9^^,^^10^.

En cuanto a la vigilancia epidemiológica nacional del resto de fuentes de infección oral de toxoplasmosis, el panorama es bastante parecido. Mediante el análisis de determinantes sociales de la salud, se sabe que el consumo de agua sin tratar es un factor de riesgo que predispone a las mujeres gestantes a la primoinfección, comparado con el consumo de agua de botella o filtrada ^11^. En otro estudio, se reportó un brote de toxoplasmosis aguda transmitida por agua en 18 militares colombianos que prestaban su servicio en el municipio de La Macarena, Meta. En ellos, se detectó como factor de riesgo epidemiológico el haber consumido agua en malas condiciones de salubridad y con presencia de sedimentos, proveniente de un cuerpo de agua estancada ubicado en el área selvática en la que prestaban su servicio ^12^.

Se han hecho estudios nacionales con técnicas moleculares para evaluar la presencia de *T. gondii* en el agua de consumo ^13^^,^^14^. En uno realizado en el departamento del Quindío, se detectó *T. gondii* en 27 de 46 (58,6 %) muestras de agua evaluadas, de las cuales 10 correspondían a agua cruda superficial, 2 a agua de la planta de tratamiento, 12 se habían recolectado en la red de distribución y 3 en los hogares ^15^. En otro estudio en Bogotá, se detectó *T. gondii* en 5 de 7 muestras de agua de grifo evaluadas provenientes de los hogares de tres mujeres gestantes positivas para IgM y cuatro gestantes negativas para IgM anti-toxoplasma (Pinzón ML, Herrera C, Gómez JE, Lora F, Arévalo A, Vanegas MC. Detección de ADN de *Toxoplasma gondii* en agua potable de Bogotá. Biomédica. 2011;31(Supl.3):241).

Por último, en un estudio en el municipio de Armenia se detectó ADN de *T. gondii* en cuatro muestras de agua provenientes de restaurantes escolares: dos de 10 muestras de agua de grifo (20 %) y dos de 30 muestras de agua hervida destinada a la elaboración de jugo de fruta (6,6 %). En este mismo estudio, se encontró que una de 12 muestras de jugo de guayaba evaluadas (8,3 %) resultó positiva por PCR para *T. gondii.* Coincidentemente, esta única muestra de jugo de fruta contaminada con ADN de *T. gondii* provenía de uno de los dos restaurantes donde la muestra de agua para la preparación de los jugos resultó positiva ^16^.

El escaso número de estudios encaminados a detectar el parásito causante de la toxoplasmosis en agua pone de manifiesto la falta de conocimiento y la subestimación del riesgo para las personas que tienen el hábito de consumir agua sin hervir o, en su defecto, agua cruda proveniente de cuerpos naturales o artificiales como los jagüeyes por falta de un sistema de abastecimiento de agua potable.

Otro agravante de la posible trasmisión de toxoplasmosis por medio del agua en Colombia, sobre todo en zonas rurales en las que no hay sistema de abastecimiento de agua potable, es que las normas vigentes se centran exclusivamente en la inspección y la vigilancia de la calidad del agua por parte de los prestadores del servicio público domiciliario de acueducto, dejando por fuera a los hogares que se abastecen de fuentes alternas como el agua lluvia, la de pozos subterráneos construidos de manera artesanal o los superficiales como los jagüeyes.

A nivel nacional, las principales normas sobre la calidad del agua son el Decreto 1575 de 2007 y la Resolución 2115 de 2007, por medio de los cuales se establece el Sistema para la Protección y Control de la Calidad del Agua para Consumo Humano. Estas mismas normas establecen que el control de la calidad del agua de los acueductos se ejerza por medio de las entidades nacionales, departamentales, municipales y distritales que forman parte del sistema, entidades que tienen la responsabilidad de velar porque los prestadores del servicio público domiciliario de acueducto cumplan con la reglamentación expedida en el marco del mismo sistema.

En el departamento de Sucre, la entidad encargada de registrar en línea los datos de las actividades de inspección, vigilancia y control de la calidad del agua para consumo humano, es el Departamento Administrativo de Seguridad Social en Salud de Sucre que, de conformidad con la Resolución 622 de 2020 emitida por el Ministerio de Salud y Protección Social y el Ministerio de Vivienda, Ciudad y Territorio, adopta el protocolo de inspección, vigilancia y control de la calidad del agua para consumo humano que suministran las entidades prestadoras del servicio público domiciliario de acueducto en la zona rural.

La mayor parte de la población rural de Sincelejo no posee acueducto, de manera que las actividades de inspección, vigilancia y control de la calidad del agua no se aplican en todo el territorio rural del municipio. Esta falencia del sistema pone en riesgo de contraer enfermedades transmitidas por consumo de agua no tratada a la población rural del municipio.

El consumo de agua sin tratar o tratada artesanalmente, tal como la proveniente de jagüeyes, podría considerarse un factor de riesgo que predispone tanto a personas como animales a contraer distintas enfermedades trasmitidas por medio del agua, entre ellas, la toxoplasmosis, debido a la posibilidad de que la escorrentía arrastre ooquistes de *T. gondii* contenidos en heces de felinos infectados.

En ese contexto, en el presente estudio se detectó ADN de *T. gondii* mediante la amplificación del gen *B1* en muestras de agua de consumo humano y agua proveniente de jagüeyes del área rural de Sincelejo, con el objetivo de establecer la relación entre la contaminación del agua por *T. gondii* y diversos determinantes sociales de la salud en el área de estudio.

## Materiales y métodos

### Tipo de estudio

Se llevó a cabo un estudio de tipo descriptivo en el que se determinó la contaminación por *T. gondii* en muestras de agua destinadas al consumo humano en hogares con acceso directo a jagüeyes y aledaños a ellos, en la zona rural del municipio de Sincelejo.

### Área de estudio

El estudio se hizo en la zona rural del municipio de Sincelejo, departamento de Sucre, ubicado al noroeste del país a 9° 18" latitud norte y 75° 23" longitud oeste del meridiano de Greenwich. Sincelejo tiene una extensión total de 248,1 km^2^, de los cuales el 92,8 % (230,24 km^2^) son territorio rural repartido en 21 corregimientos. La temperatura media anual del municipio está cercana a los 32 °C, con una mínima de 28 °C y una máxima de 38 °C.

**Figura 1:**
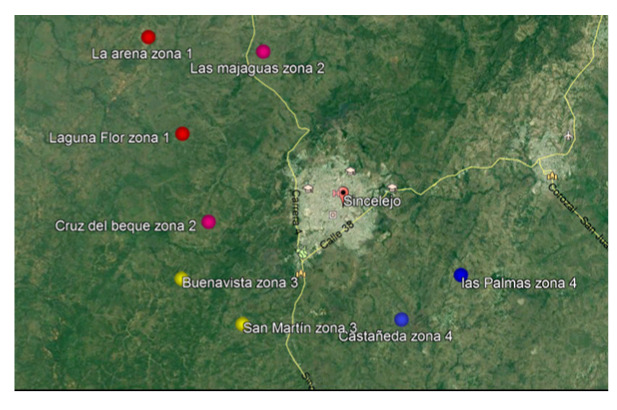
Zonas y corregimientos de muestreo

Sincelejo posee 300 jagüeyes, la mayoría distribuidos en la zona rural del municipio ^17^. En este estudio, se analizaron muestras de agua provenientes de 48 de ellos (16 %) ubicados en ocho corregimientos de la zona rural de Sincelejo, los cuales están agrupados es cuatro zonas geoeconómicamente similares según el Plan de Ordenamiento Territorial. Los corregimientos evaluados por zonas fueron: zona 1: La Arena y Laguna Flor; zona 2: Las Majaguas y Cruz del Beque; zona 3: Buenavista y San Martín, y zona 4: Castañeda y Las Palmas (figura 1).

Nota: cada par de corregimientos tiene asignado un color según la zona de estudio. Fuente: modificado de Google Earth

La selección de estos corregimientos y de los jagüeyes examinados se hizo mediante un muestreo no probabilístico por conveniencia. Los criterios de selección fueron la cercanía del jagüey a la vía de acceso, lo cual se verificó con un recorrido virtual superficial del área del municipio de Sincelejo utilizando el programa Google Earth Pro 7.3.3.7786, y la accesibilidad al cuerpo de agua permitida por los habitantes del predio.

### Tamaño de la muestra

Se recolectaron 96 muestras de agua para consumo humano y labores domésticas. De estas 96 muestras de agua, 48 eran agua cruda de jagüey y 48 eran de agua tratada artesanalmente para el consumo directo. En cada corregimiento se tomaron 12 muestras de agua, seis de agua cruda proveniente de jagüeyes y seis tratadas artesanalmente de los hogares aledaños a los jagüeyes, cada una con un volumen de 5 litros ^18^. Las muestras se almacenaron en recipientes plásticos estériles y se transportaron desde el sitio de muestreo hasta el Laboratorio de Investigaciones Biomédicas de la Universidad de Sucre, para su análisis.

*Concentración de las muestras.* Las muestras de agua se sometieron a un proceso previo de filtración con papel de filtro comercial (filtros de café) para eliminar partículas de gran tamaño. Luego se pasaron a través de membranas de celulosa con un tamaño de poro de 5 μm en un equipo de filtración formado por una bomba de vacío, un papel filtro, un embudo y un matraz recolector de Erlenmeyer. Las muestras de agua cruda de jagüey se filtraron en dos volúmenes, cada uno de 2,5 litros, utilizando una membrana de celulosa para cada volumen. Las membranas de celulosa empleadas en la filtración se dispusieron en tubos Falcon™ de 15 ml para la posterior elución de ooquistes y la extracción de ADN.

### Elución de ooquistes

Esta se hizo con el método descrito por Isaac-Renton, *et al.,* con algunas modificaciones ^19^. Los filtros se lavaron con 10 ml de PBS complementado con Tween 80 al 0,01 % y SDS al 0,01 % en tubos Falcon™ de 15 ml. Los tubos se colocaron en un agitador durante 30 minutos y después los filtros fueron retirados del tubo; la solución obtenida fue centrifugada a 4.500*g* durante 10 minutos. El gránulo obtenido se resuspendió en 10 ml de agua destilada y fue nuevamente centrifugado a 4.500g durante 10 minutos.

Los ooquistes de *T. gondii* se recuperaron mediante el método de flotación con solución azucarada usado por Sroka ^18^, con algunas modificaciones. El gránulo obtenido a partir de la elución fue resuspendido en 3 ml de solución de sacarosa compuesta por 53 g de sacarosa, 100 ml de agua destilada y 0,8 ml de fenol (gravedad específica de 1,15). Los tubos se centrifugaron y el sobrenadante fue recuperado y adicionado a un nuevo tubo Falcon™ en el que, posteriormente, se adicionaron 9 ml de PBS complementado con Tween 80 al 0,01 % y SDS al 0,01 %. Después de una última centrifugación, el gránulo (ooquistes) fue resuspendido en 500 μl de solución tampón TE y almacenado en un tubo Eppendorf de 1,5 ml.

### Extracción de ADN genómico

La extracción de ADN se llevó a cabo mediante el método de fenol-cloroformo.

### Amplificación del control interno positivo

Como control interno positivo, se amplificó ADN de bacterias, específicamente el gen *ARNr 16s,* con los cebadores 27F y 1492R, los cuales amplifican un fragmento de 1.500 pb. La mezcla de reacción estuvo conformada por 5 μl de solución tampón 5X incoloro, 1,5 μl de MgCl_2_, 0,5 μl de DNTP, 0,125 μl de GoTaq™, 2 μl de ADN, 15,275 μl de agua ultrapura y 0,3 μl de cada iniciador para un volumen de reacción de 25 μl. El perfil térmico incluyó una etapa inicial de desnaturalización a 95 °C durante dos minutos, de desnaturalización a 95 °C durante 30 segundos, un alineamiento a 50 °C durante 30 segundos, una extensión a 72 °C durante un minuto y una extensión final a 72 °C durante cinco minutos. Se usó como control positivo ADN de rickettsias y, como control negativo, un volumen de agua ultrapura.

### Amplificación del gen B1

Para la amplificación de la región del gen *B1* de *T. gondii,* se usó la PCR anidada con dos conjuntos de cebadores descrita previamente (20). El proceso de amplificación se hizo en dos etapas; una primera ronda de amplificación de un fragmento de 193 pb con los cebadores toxo N1 y toxo C1, y una segunda mediante la amplificación de un fragmento de 96 pb con los cebadores toxo N2 y toxo C2. Los pares de cebadores empleados fueron sintetizados con la plataforma de Integrated DNA Technologies IDT™ y el proceso de amplificación se llevó a cabo en un termociclador Veriti 96-Well Fas™ (Applied Biosystems).

La mezcla de reacción de la primera ronda se compuso de 5 μl de solución tampón 5X incolora, 1,5 μl de MgCl_2_, 0,5 μl de DNTP, 0,125 μl de GoTaq™, 3 μl de ADN, 14,275 μl de agua ultrapura y 0,3 μl de cada iniciador, para un volumen de reacción de 25 μl. El perfil térmico de la primera ronda de amplificación incluyó una etapa inicial de desnaturalización a 95 °C durante dos minutos, una de desnaturalización a 95 °C durante 30 segundos, un alineamiento a 54 °C durante 30 segundos, una extensión a 72 °C durante 45 segundos y una extensión final a 72 °C durante cinco minutos. Como control positivo se usó ADN de *T. gondii* y, como control negativo, un volumen de agua ultrapura.

La mezcla de reacción de la segunda ronda de amplificación se compuso de 5 μl de solución tampón 5X incolora, 1,5 μl de MgCl_2_, 0,5 μl de DNTP, 0,125 μl de GoTaq™, 3 μl de ADN (amplicón de primera ronda), 13,675 μl de agua ultrapura y 0,6 μl de cada iniciador, para un volumen de reacción de 25 μl. El perfil térmico de la segunda ronda de amplificación incluyó una etapa inicial de desnaturalización a 95 °C durante dos minutos, una de desnaturalización a 95 °C durante 30 segundos, un alineamiento a 54 °C durante 30 segundos, una extensión a 72 °C durante 30 segundos y otra final a 72 °C durante cinco minutos. Como control positivo se usó el producto de amplificación de la primera ronda del control positivo y, como control negativo, el producto de amplificación de la primera ronda del control negativo.

### Visualización de los productos de la PCR

Los productos de la PCR se separaron mediante una electroforesis en gel de agarosa al 1,5 % en solución tampón TBE 0,5X (tris-ácido bórico-EDTA) a 80 voltios durante 40 minutos. Los resultados se visualizaron en un fotodocumentador después de teñirlos con Gelstar®. Se utilizó un marcador de peso molecular de 100 pb, con el fin de determinar el tamaño de los fragmentos amplificados.

### Análisis de los determinantes sociales de la salud

Los determinantes sociales de la salud en los 48 lugares de muestreo se evaluaron mediante una encuesta compuesta por 15 preguntas para recolectar la información relacionada con cuatro de ellos: las características físicas de la vivienda, la presencia de gatos, la disponibilidad del agua y sus usos. Los resultados obtenidos en las encuestas se ingresaron en una base de datos diseñada en Excel 2013 y sirvieron para evaluar su asociación con la contaminación del agua por *T. gondii.*

### Análisis estadístico de los datos

Con base en los resultados obtenidos de las pruebas de laboratorio y las respuestas a la encuesta, se evaluó la asociación entre la contaminación del agua por *T. gondii* y los determinantes sociales de la salud analizados.

La asociación entre variables dicotómicas se evaluó mediante el test de Fisher con una tabla de contingencia 2 x 2. La asociación entre la contaminación del agua por *T. gondii* y los determinantes sociales de la salud con más de dos respuestas categóricas, se evaluó con el test exacto de Fisher y el paquete estadístico "fmsb library" Los dos análisis estadísticos se hicieron con el programa estadístico R™, versión 3.3.2.

## Resultados

### Características del agua recolectada y los sitios de muestreo

Las 96 muestras de agua recolectadas se caracterizaron por presentar baja salinidad, poca conductividad eléctrica y pH neutro (no se muestran los datos). Todas las muestras tenían poca turbidez, aunque las de agua cruda de jagüey eran ligeramente más turbias, comparadas con las tratadas artesanalmente. Por ello, fue necesario filtrarlas separadamente en dos volúmenes de 2,5 litros cada uno y emplear para cada volumen una membrana de celulosa diferente.

**Figura 2:**
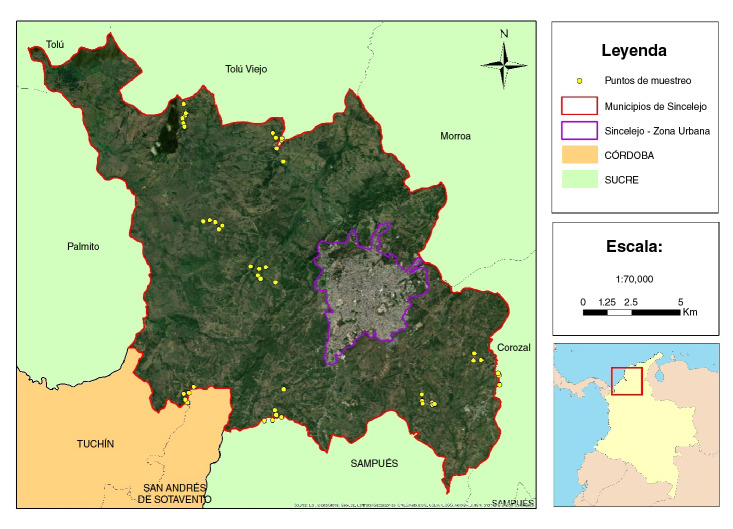
Ubicación de los lugares de muestreo

Los 48 sitios de muestreo se clasificaron como fincas o pequeñas parcelas ubicadas en la zona rural de Sincelejo (figura 2). Todos pertenecían al estrato social 1 y las viviendas estaban construidas en concreto (n=28), bahareque (n=10) y madera (n=1), así como con materiales mixtos (n=9). Todas las viviendas contaban con el servicio de electricidad y solo 10 tenían servicio de acueducto, por lo que la mayoría se abastecía con agua proveniente de los jagüeyes.

Fuente: elaborada en ArcGIS

### Amplificación del control interno positivo

Para evaluar la integridad del ADN extraído, se hizo una prueba de amplificación de control interno positivo usando como molde el ADN extraído a partir de las 96 muestras de agua que fueron filtradas, con el fin de amplificar material genético de bacterias, específicamente un fragmento del gen *ARNr 16S.* Se consideraron positivas aquellas muestras a partir de las cuales se habría amplificado un fragmento de 1.500 pb de dicho gen.

De las 96 muestras analizadas, 90 amplificaron el fragmento deseado para la prueba de control interno de amplificación. En las seis restantes, fue necesario diluir 1/100 su ADN para lograr tal amplificación. Todas las muestras amplificaron para el control interno positivo (100 %) y se evaluaron molecularmente para determinar si estaban o no contaminadas con ADN de *T.gondii.*

### Detección molecular de contaminación por T. gondii

*Amplificación del gen B1.* Para la prueba de PCR anidada se utilizó como molde el ADN extraído de las 96 muestras de agua recolectadas en la zona rural del municipio de Sincelejo, con el fin de amplificar un fragmento del gen *B1* de *T. gondii.* Las muestras se consideraron positivas cuando, a partir de su ADN total, se habría amplificado un fragmento de 96 pb en la etapa anidada de la PCR. Fue posible detectar ADN de *T. gondii* en 13 de 96 (13.5%) muestras analizadas mediante PCR anidada, luego de visualizar en gel de agarosa los productos de amplificación.

### Detección de Toxoplasma gondii por tipo de muestra

De las 13 muestras de agua que resultaron positivas para contaminación por *T.gondii* (13,5 %), nueve correspondían a muestras de agua cruda de jagüeyes (18,8 %) y cuatro a muestras de agua tratadas artesanalmente (8,3 %). En los análisis estadísticos (test de Fisher), se encontró que la contaminación con *T.gondii* no dependió del tipo de agua analizada (p=0,2328) (cuadro 1).

**Cuadro 1:** Detección de Toxoplasma gondii por tipo de muestra y lugares de recolección

Variable	Muestras evaluadas	Muestras positivas	%	IC_95%_	χ^2^ (p)
Tipo de muestra					
Superficial	48	9	18,75	9,4 - 33,10	
Tratada artesanalmente	48	4	8,33	2,70 - 20,87	1,4235 (0,2328)
Zona de recolección					
1	24	1	4,17	0,22 - 23,12	
2	24	5	20,83	7,94 - 42,71	
3	24	6	25,00	10,60 - 47,05	
4	24	1	4,17	0,22 - 23,12	7,3846 (0,0606)
Corregimiento					
Laguna Flor	12	0	0,00	0 - 30,13	
La Arena	12	1	8,33	0,44 - 40,25	
Cruz del Beque	12	2	16,67	2,94 - 49,12	
Las Majaguas	12	3	25,00	6,69 - 57,16	
Buenavista	12	3	25,00	6,69 - 57,16	
San Martín	12	3	25,00	6,69 - 57,16	
Castañeda	12	0	0,00	0 - 30,13	
Las Palmas	12	1	8,33	0,44 - 40,25	8,4523 (0,2944)

### Detección de Toxoplasma gondii por lugares de estudio

En cuanto a los lugares de recolección de las muestras, se encontró que la zona con mayor número de muestras contaminadas por *T.gondii* fue la 3, conformada por los corregimientos de Buenavista y San Martín, con seis muestras positivas (25 %), seguida por la zona 2 (Las Majaguas y Cruz del Beque), con cinco muestras positivas (20,8 %), en tanto que las zonas 1 (Laguna Flor y La arena) y 4 (Castañeda y Las Palmas) registraron una muestra positiva (4,2 %) cada una. El análisis estadístico no mostró asociación entre la contaminación por *T.gondii y* las zonas de muestreo (p=0,0606).

**Figura 3:**
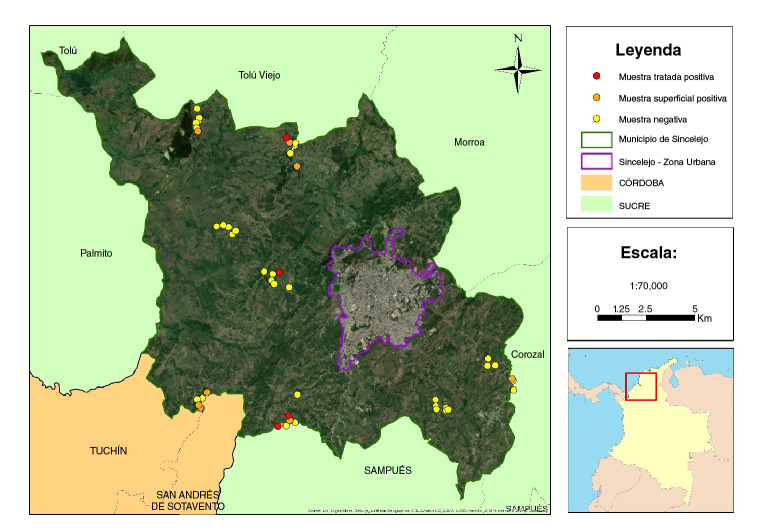
Distribucion espacial de muestras de agua contaminadas con *Toxoplasma gondii*

Con respecto a los corregimientos, no se encontró una diferencia estadística en el número de muestras positivas para *T.gondii* por corregimiento (p=0,2944). Los corregimientos con mayor número de muestras positivas fueron Buenavista, Las Majaguas y San Martín, cada uno con tres, seguidos por Cruz del Beque con dos, y por La Arena y Las Palmas, cada uno con una muestra positiva (cuadro 1 y figura 3).

Fuente: elaborada en ArcGIS

### Asociación de la positividad con los determinantes sociales de la salud

Dichos factores en los 48 lugares de muestreo se evaluaron mediante una encuesta que recopiló la información relacionada con las características físicas de la vivienda, la presencia de gatos, la disponibilidad del agua y sus usos. Dicha información sirvió para evaluar la asociación entre la contaminación del agua por *T. gondii* y los mencionados determinantes sociales de la salud, mediante un test de Fisher realizado en el programa estadístico R™, versión 3.3.2.

En cuanto a las características físicas de las viviendas, se encontró que la mayoría era de concreto (58,3 %), seguida de las de bahareque (20,8 %), y las de construcción mixta (18,8 %), es decir, con al menos dos de los materiales de construcción mencionados. El piso del 52,1 % de las viviendas era de plantilla de cemento, el 25 % tenía piso de arena, el 10,4 %, piso de cerámica y, el 12,5 %, piso de materiales mixtos (cuadro 2).

**Cuadro 2:** Muestras contaminadas con *Toxoplasma gondii* según las características físicas de la vivienda

Variable	Fincas encuestadas	Muestras positivas		IC_95%_	x^2^ (p)
		(n)	%		
Material de la vivienda					
Bahareque	10	4	40	13,69 - 72,63	
Concreto	28	7	25	11,43 - 45,22	
Madera	1	0	0	0,00 - 94,54	
Mixto	9	2	22	3,95 - 59,81	1,3855 (0,7089)
Material del piso de la vivienda					
Arena	12	6	50	25,38 - 74,62	
Cemento	25	5	20	7,61 - 41,30	
Cerámica	5	2	40	7,26 - 82,96	
Mixto	6	0	0	0,00 - 48,32	6,4774 (0,09056)

Al analizar el número de muestras positivas según estas dos variables, se encontró que las casas de concreto y aquellas con piso de arena registraron el mayor número de muestras positivas para contaminación con *T. gondii,* cada una con siete y seis muestras positivas, respectivamente. Sin embargo, el análisis estadístico indicó que la contaminación por *T. gondii* en el agua de consumo humano proveniente de jagüeyes no dependió del material de construcción de la vivienda ni del tipo de piso que tuvieran (p>0,05).

En cuanto a la presencia de gatos, se encontró que en 27 de las 48 fincas o parcelas visitadas había gatos como mascotas (56,25 %), pero en ellas solo se encontraron seis muestras positivas correspondientes a cuatro de agua superficial y a dos de agua tratada artesanalmente. La mayoría de las fincas en las que había gatos como mascota tenía máximo dos gatos (16 fincas uno, y ocho, dos) y solo en tres fincas se encontraron entre cuatro y nueve gatos. En las fincas donde solo había un gato se encontró el 50 % (tres de seis) de las muestras positivas para contaminación con *T. gondii.* Otro dato importante es que la finca con nueve gatos fue la única con las dos muestras positivas para contaminación por *T. gondii.*

En cuanto a la edad de los gatos, en la mayoría (12 de 27) de las fincas estos eran adolescentes, con edades entre los siete meses y los dos años de edad, y en ellas se reportaron la mayoría de las muestras de agua positivas para contaminación por *T. gondii* (5 de 6). De las 48 fincas encuestadas, solo en 12 había contacto de la familia con los gatos, en 26 se recogían las heces de las mascotas y en 16 merodeaban los gatos callejeros. El número de muestras positivas para cada una de estas variables de estudio difirió; sin embargo, al evaluar la dependencia de la contaminación del agua por *T. gondii* con cada una de las variables relacionadas con la presencia de gatos, no se encontró una asociación estadística (p>0,05). Dicho de otra manera, la contaminación por *T. gondii* en agua de jagüeyes y agua tratada artesanalmente en la zona de estudio no dependió de la presencia de gatos en las viviendas ni de los hábitos de higiene practicados en estos animales (cuadro 3).

**Cuadro 3:** Muestras contaminadas con *Toxoplasma gondii* según la presencia de gatos

Variable	Fincas encuestadas	Muestras positivas		IC_95%_	χ^2^ **(p)**
		(n)	(%)		
Presencia de gatos					
Sí	27	6	22,22	9,38 - 42,73	
No	21	7	33,33	15,48 - 56,89	0,28299 (0,5947)
Número de gatos					
1	16	3	18,75	4,97 - 46,31	
2	8	1	12,50	0,66 - 53,32	
4	1	0	0	0,00 - 94,54	
8	1	2	100	5,46 - 100	
9	1	0	0	0 - 94,54	5,0472 (0,2825)
Edad de los gatos					
Bebés	9	1	11,11	0,58 - 49,33	
Adolescentes	12	5	41,67	16,50 - 71,40	
Jóvenes maduros	5	0	0	0,00 - 53,71	
Adultos	1	0	0	0,00 - 94,54	4,9821 (0,1731)
Contacto con gatos					
Sí	12	3	25	6,69 - 57,16	
No	36	10	27,78	14,79 - 45,43	4,5771e-31 (1)
Presencia de gatos callejeros					
Sí	16	4	25	8,33 - 52,59	
No	32	9	28,13	14,40 - 46,98	4,2147e-31 (1)
Recoge las heces					
Sí	26	8	30,77	15,09 - 51,90	
No	22	5	22,73		0,089265 (0,7651)

Con respecto a las variables relacionadas con el agua, se encontró que el 33,3 % de las fincas visitadas utilizaban el jagüey y otra fuente externa para abastecerse de agua (16 de 48 tenían suministro mixto). Se encontró, también, que solo 10 (20,8 %) viviendas estaban conectadas al suministro de agua potable de acueducto, en tanto que otras diez (20,8 %) se abastecían con agua transportada en carrotanques. En cuanto a la periodicidad del suministro de agua, la mayoría de las viviendas contaba con agua la mayor parte de los días del año, no porque estuvieran conectadas al acueducto, sino porque utilizaban diferentes fuentes para autoabastecerse. En el caso de las viviendas con servicio de acueducto, la mayoría tenía suministro de agua cada ocho días (cinco viviendas), otras todos los días (tres viviendas) y otras cada dos días (dos viviendas). En cuanto al lugar de almacenamiento del agua, el 58,3 % (28 de 48) de las viviendas encuestadas la almacenaba en tanques plásticos o de cemento, el 16,6 % tenía almacenamiento mixto, el 14,6 % en alberca de cemento, 8,3 % en alberca subterránea y 2 % en tinas. Los tests de Fisher para comparar la contaminación del agua con *T. gondii* con las variables relacionadas con el agua, no evidenciaron dependencia entre dichas variables (p>0,05) (cuadro 4).

**Cuadro 4:** Muestras contaminadas con *Toxoplasma gondii* según el tipo de agua

Variable	Fincas encuestadas	Muestras positivas		IC_95%_	χ^2^ **(p)**
		(n)	(%)		
Suministro de agua					
Acueducto	10	1	10	0,52 - 5,88	
Carrotanque	10	3	30	8,09 - 64,63	
Jagüeyes	4	2	50	15,00 - 85	
Lluvia	5	3	60	17,04 - 92,7	
Mixto	16	3	18,75	4,97 - 46,31	
Pimpinas	2	1	50	9,45 - 90,55	
Pozo subterráneo	1	0	0	0 - 94,54	6,7938 (0,3403)
Periodicidad del suministro de agua					
Todos los días	16	5	31,25	12,13 - 58,5	
De 2 a 8 días	13	4	30,77	10,36 - 61,1	
Cada 15 días	3	0	0	0 - 69	
Cada 2 meses	3	0	0	0 - 69	
Cada 6 meses	1	0	0	0 - 94,54	
Anual	1	1	100	5,46 - 100	
Nunca	11	3	27,27	7,33 - 60,68	5,5226 (0,4787)
Lugar de almacenamiento del agua					
Alberca de cemento	7	3	42,86	11,81 - 79,7	
Alberca subterránea	4	0	0	0 - 60,42	
Tanques	28	7	25	11,43 - 45,2	
Tinas	1	0	0	0 - 94,54	
Mixto	8	3	37,5	10,24 - 74,1	3,2402 (0,5185)

En cuanto a los usos del agua, se encontró que la mayoría de las viviendas (31 de 48: 64,6 %) consumía agua cruda, lo que evidencia el riesgo al que podría estar expuesta la población. El resto de viviendas consumía agua tratada artesanalmente (hervida, filtrada, en bolsa). Sin embargo, el análisis estadístico no evidenció dependencia entre la contaminación del agua por *T. gondii* y el tipo de agua consumido en la zona de estudio (p=0,2722), lo que indica que el consumo de agua tratada no sería un hábito que evite contraer la toxoplasmosis.

El tipo de agua más utilizada para el lavado de los alimentos, así como para su preparación, era la suministrada por el acueducto (16 de 48 viviendas en cada ítem, es decir, 33,33 % por ítem). Es importante aclarar que, aunque no todas las fincas incluidas en el estudio contaban con este tipo de suministro, se encontraron viviendas con suministro mixto que utilizaban el agua del jagüey para los oficios diarios y, el agua transportada en pimpinas desde Sincelejo (que sí cuenta con acueducto de agua potable), para los alimentos. El uso de agua potable para el lavado y la preparación de alimentos no podría considerarse un hábito protector contra la toxoplasmosis en la zona de estudio, ya que según el test de Fisher, la contaminación con *T. gondii* no dependió del tipo de agua utilizado para lavar y preparar los alimentos (p>0,05).

En cuanto al agua para el riego de cultivos, el 31 (64,6 %) de las fincas aprovechaban exclusivamente las temporadas de lluvia para regar sus cultivos, en tanto que las 14 fincas restantes usaban de manera alternada el agua lluvia y el agua proveniente de los jagüeyes.

En el test Fisher para establecer la asociación entre la contaminación por *T. gondii* y el tipo de agua para el riego de cultivos, no se encontró dependencia alguna (p=1), de manera que ninguno de los sistemas de riego podría haber incidido en la contaminación del agua por *T. gondii* en la zona de estudio (cuadro 5).

**Cuadro 5:** Muestras contaminadas con *Toxoplasma gondii* según los usos del agua

Variable	Fincas encuestadas	Muestras positivas		IC_95%_	x^2^ (p)
		(n)	(%)		
Tipo de agua para consumo					
Cruda	31	9	29,03	14,8 - 48,2	
En bolsa	8	1	12,5	0,66 - 53,3	
Filtrada	1	1	100	5,46 - 100	
Hervida	5	2	40	7,26 - 82,9	
Mixta	3	0	0	0 - 69	5,1502 (0,2722)
Agua para el lavado de alimentos					
Acueducto	16	5	28,57	9,58 - 58	
Alberca de cemento	1	0	0	0 - 94,54	
Carrotanque	7	1	14,29	0,75 - 57,9	
En bolsa	1	0	0	0 - 94,54	
Jagüeyes	4	2	50	15,00 - 85	
Lluvia	7	3	42,86	11,8 - 79,7	
Pozo subterráneo	1	0	0	0 - 94,54	
Mixta	11	2	18,18	3,21 - 52,2	4,2225 (0,7538)
Agua para la preparación de los alimentos					
Acueducto	16	5	28,57	9,58 - 58	
Alberca de cemento	1	0	0	0 - 94,54	
Carrotanque	7	1	14,29	0,75 - 57,9	
En bolsa	1	0	0	0 - 94,54	
Jagüeyes	3	2	66,67	12,5 - 98,2	
Lluvia	10	4	40	13,7 - 72,6	
Pozo subterráneo	1	0	0	0 - 94,54	
Mixta	9	1	11,11	0,58 - 49,3	6,2232 (0,5139)
Agua para el riego de los cultivos					
Lluvia	34	9	26,47	13,5 - 44,7	
Mixta	14	4	33,33	6 - 75,89	2, 3712e-31 (1)

## Discusión

*Toxoplasma gondii* es el protozoo parásito intracelular obligado causante de una de las principales enfermedades zoonóticas a nivel mundial. Tiene un ciclo de vida que involucra tres formas infectivas: los taquizoítos, los quistes tisulares que contienen bradizoítos, y los ooquistes que contienen esporozoítos. Los ooquistes son extremadamente resistentes, ya que pueden sobrevivir durante meses en el medio ambiente e infectar a los humanos por medio del consumo de agua contaminada o no tratada; por ello, el agua se considera un posible vehículo de diseminación de la toxoplasmosis.

A nivel mundial, se han reportado varios brotes de toxoplasmosis transmitida por el consumo de agua no filtrada y contaminada con ooquistes. El primer brote de toxoplasmosis aguda asociada al consumo de agua ocurrió en 1979, cuando 35 soldados de un batallón estadounidense entrenados en una jungla de Panamá consumieron agua de dos fuentes naturales localizadas en el lugar de entrenamiento ^21^. El segundo brote de toxoplasmosis aguda asociada al agua de consumo, ocurrió en 1995 en un área de Gran Victoria, Columbia Británica, población que se abastecía de una planta de desinfección que suministraba agua clorada, no filtrada, proveniente del reservorio Humpback ^22^. Una investigación posterior logró demostrar que el agua de aquel reservorio estaba contaminada con ooquistes de *T. gondii* presentes en las heces de los pumas que habitaban en el área. También, se encontró evidencia serológica de toxoplasmosis en gatos domésticos de Gran Victoria, con lo cual se concluyó que se había ocurrido un ciclo de toxoplasmosis endémica en el área ^23^.

Asimismo, en Santa Isabel do Ivai, en el estado de Paraná, Brasil, se documentó un brote de toxoplasmosis asociado al consumo de agua contaminada. El brote ocurrió entre noviembre de 2001 y enero de 2002; se reportaron 294 casos de toxoplasmosis aguda y se logró determinar que las personas habían consumido agua o helados preparados con agua de un reservorio presumiblemente contaminado con ooquistes de *T. gondii,* pues se encontró una gata que había vivido en la parte posterior del reservorio y en ese mismo lugar había tenido su cría hasta el momento del destete. Sin embargo, no se logró determinar serológicamente la toxoplasmosis en los felinos debido a que no pudieron ser capturados ^24^.

Entre el 2004 y el 2005, se reportó en India un brote de toxoplasmosis ocular adquirida en 248 pacientes. De ellos, 209 vivían en la ciudad de Coimbatore, razón por la cual se dedujo que todos habían utilizado el agua de la ciudad, cuyo aprovisionamiento provenía de pequeños ríos y corrientes que descendían de las colinas en el norte y el este de la ciudad. Se presumió que el agua usada por los pacientes era la fuente más probable de la infección con toxoplasmosis, aunque no se pudo determinar con precisión debido a que no se presentaron casos clínicos similares en el segundo semestre de 2005 ^25^.

En el 2009, en Colombia, se reportó un brote de toxoplasmosis aguda asociada al consumo de agua en 18 soldados que prestaban su servicio militar en el municipio de La Macarena, departamento del Meta. Se estableció, como posible factor de riesgo epidemiológico, el consumo de agua en malas condiciones y presencia de sedimentos provenientes de un cuerpo de agua léntico ubicado en el área selvática en la cual prestaban su servicio ^12^.

En el presente estudio, se detectó ADN de *T. gondii* mediante una PCR anidada en el 13,5 % de las muestras de agua de consumo humano provenientes de la zona rural del municipio de Sincelejo, así: agua cruda proveniente de jagüeyes (18,8 %) y agua tratada artesanalmente para el consumo directo (8,3 %). Aunque el porcentaje de muestras positivas por PCR fue bajo, y esta prueba no demuestra capacidad infectiva, los resultados positivos podrían ser un indicador del nivel de exposición del agua para consumo humano a protozoos patógenos como *T. gondii.* Asimismo, los resultados negativos para *T. gondii* no deben entenderse como indicadores de una baja exposición al riesgo de contraer toxoplasmosis por medio del agua, ya que es posible que estén más relacionados con la presencia de inhibidores de la PCR en muestras ambientales en las que se dificulta la extracción de ADN pues, como lo han señalado otros autores, la pared de los ooquistes de los protozoos es un blanco difícil para los métodos de extracción de ácidos nucleicos ^15^.

En la zona del estudio no había acceso directo a agua potable por no tener acueducto. Solo un corregimiento de los ocho visitados contaba con este servicio, por lo que la mayoría de los hogares se abastecían en fuentes alternas, como los jagüeyes, la lluvia, los pozos subterráneos e, incluso, agua no potable transportada en carrotanques. Estos determinantes sociales de la salud, sumados a la contaminación con *T. gondii* del agua, demuestran el potencial riesgo de contraer toxoplasmosis al que está sometida la población rural de Sincelejo.

En cuanto al tipo de agua analizada, las muestras de agua cruda de jagüey presentaron una turbidez ligeramente mayor a la de las muestras de agua tratada artesanalmente. Ambos tipos de muestras se sometieron al mismo proceso de concentración, pero las muestras de agua cruda de jagüey tuvieron que filtrarse separadamente en dos volúmenes, cada uno de 2,5 litros, lo que sugiere que las membranas de celulosa con un tamaño de poro de 5 μm dificultan el proceso de filtración de grandes volúmenes de agua, especialmente si la muestra trae gran cantidad de material en partículas, pero son útiles para retener partículas que superen el tamaño del poro, tal como los quistes de *T. gondii,* los cuales miden aproximadamente 12 μm.

Debido a la porosidad de los filtros usados en este estudio, es muy probable que se hayan obtenido también quistes de otros parásitos transmitidos por el agua, tales como *Giardia* spp., *Cryptosporidium* spp. e, incluso, bacterias (en forma de agregados), que no fueron evaluadas en este estudio, pero que pudieron haber obstruido la membrana microporosa, impidiendo el paso del volumen total de la muestra.

En cuanto a la contaminación con *T. gondii* por tipo de muestra, se encontró que nueve de 48 (18,8 %) muestras superficiales resultaron contaminadas con *T. gondii,* en tanto que solo en cuatro de las 48 (8,3 %) muestras de agua para consumo directo se encontró el parásito. A pesar de que el análisis no evidenció diferencias estadísticas, estos resultados permiten inferir que había más probabilidad de detectar el parásito causante de la toxoplasmosis en el agua superficial que en la almacenada en tanques herméticamente tapados o con algún tipo de seguridad.

Estos resultados son similares a los encontrados en un estudio llevado a cabo en la provincia de Lublin, Polonia, en el que se encontraron 31 (27,2 %) muestras de agua positivas para *T. gondii* de un total de 114 evaluadas por PCR. De estas 31 muestras positivas, 30 correspondían a agua de pozos poco profundos, y solo una a agua de un pozo profundo. Los autores señalan que las muestras de agua de los pozos poco profundos se tomaron al nivel más superficial y que dichos pozos, además, no se encontraban cubiertos por capas impermeables ^18^.

Nuestros resultados también se asemejan a los reportados en el noreste de Polonia, en donde se encontró que siete de 36 (19,4 %) muestras de agua de fuentes superficiales resultaron positivas para *T. gondii* por PCR. Esas siete muestras provenían de cuatro lagos, dos ríos y un estanque ^26^. En otro estudio en Serbia, tres de 20 muestras de agua de río resultaron positivas para *T. gondii* por PCR. Los lugares de muestreo que resultaron positivos estaban situados, uno en el Danubio y dos en el Drina ^27^.

Por ser cuerpos de agua superficial, los jagüeyes están permanentemente expuestos a recibir material contaminante. Por acción de la escorrentía pueden llegar hasta ellos ooquistes de *T. gondii* excretados por felinos infectados, lo que aumenta el riesgo de transmitir el parásito a quienes consumen dicha agua sin ningún tratamiento de desinfección. En los hogares incluidos en este estudio, el agua tratada artesanalmente y destinada al consumo directo se almacenaba en tanques tapados, lo que reduce el riesgo de contaminación y podría explicar la menor frecuencia de detección de *T. gondii* en este tipo de agua.

En un estudio en São Paulo, Brasil, se encontró que tres de 39 (7,7 %) muestras de agua superficial de ríos resultaron positivas para *T. gondii.* Todas se habían recolectado durante la época de lluvia, lo que según los investigadores indicaría una mayor presencia de contaminantes en los cuerpos de agua por acción de la escorrentía ^28^.

En cuanto a la frecuencia de detección de *T. gondii* por sectores de la zona de estudio, se encontró que aquellos con mayor número de muestras contaminadas fueron las zonas 3 y 2, conformadas por los corregimientos de Buenavista y San Martín, y Las Majaguas y Cruz del Beque, respectivamente. Aunque no hubo diferencia estadística entre el número de muestras positivas en estas zonas y el de las zonas 1 y 4, los resultados podrían asociarse con diferentes determinantes sociales de la salud, entre ellos la presencia de acueducto. El corregimiento de Castañeda, perteneciente a la zona 4, es el único corregimiento que posee acueducto, lo que mejora la calidad de vida de sus habitantes con el suministro de agua potable.

Para efectos del estudio, el servicio de acueducto en Castañeda implicaría una menor probabilidad de encontrar ADN de *T. gondii,* pues el tratamiento del agua habría eliminado restos biológicos del parásito. También debe señalarse que en las zonas 2 y 3 se encontró un mayor número de muestras de agua superficial positivas para *T. gondii* (7 de 9) que en las zonas 1 y 4 (2 de 9), lo que podría relacionarse con la presencia de felinos silvestres, lo cual no se evaluó en nuestro estudio.

En esta zona se ubican, entre otros, los corregimientos de San Martín y Buenavista, en los cuales se encuentran asentamientos indígenas cuya cultura está ligada a la preservación del medio ambiente ^29^. Además, según el Plan de Conservación de Felinos del Caribe Colombiano, se ha reportado la presencia de dos especies de felinos silvestres, *Leopardus pardalis* y *Leopardus wiedii,* en la subregión de Montes de María, y su distribución espacial se asocia con el estado de conservación del paisaje natural, de manera que los felinos son más abundantes en hábitats con menos intervención antrópica ^30^.

Por último, es posible que los felinos silvestres que circulan en la zona 3 hayan sido infectados por "carnivorismo" (sic) (forma de alimentación que permite obtener nutrientes a partir del consumo de carne o de animales presas), y sus heces podrían ser la fuente de contaminación con *T. gondii* de los cuerpos de agua superficial en esta zona. Sin embargo, en este estudio no se evaluó la seroprevalencia en felinos ni se detectaron parásitos en sus heces. En el estudio en Serbia se encontraron tres puntos de muestreo del río positivos para *T. gondii,* lo que según los investigadores podría relacionarse con la presencia de felinos. En el caso del Danubio, que pasa por una zona urbana, los gatos callejeros, asilvestrados o domésticos que ocasionalmente dejan sus hogares, serían la más probable fuente de ooquistes. En el caso del río Drina, este pasa por una zona rural próxima a los bosques serbios que alberga las especies de felinos silvestres lince *(Lynx Lynx)* y gato montés *(Felis silvestris),* los cuales podrían ser responsables de los ooquistes encontrados en el río ^27^.

En el presente estudio, se evaluó la relación entre cuatro determinantes sociales de la salud (características físicas de la vivienda, presencia de gatos, disponibilidad del agua y sus diferentes usos) y la frecuencia de detección de *T. gondii* en el agua. No se encontró ninguna relación estadística entre ellos, lo que quiere decir que en la zona de estudio dichos factores no inciden en la contaminación del agua con *T. gondii.* Es posible que allí estén interviniendo otros factores intermedios que no fueron evaluados en este estudio, como la cubierta de los tanques de almacenamiento del agua, la frecuencia con que se limpian y desinfectan, la disposición de las basuras, la frecuencia de la limpieza del patio de las fincas, la infestación por ratones y la presencia de otros animales domésticos, los cuales podrían ayudar a mantener activa la circulación de la toxoplasmosis en la zona.

Un dato importante encontrado en esta parte del estudio es que los determinantes sociales estudiados demuestran que la zona de estudio presenta muchas necesidades básicas insatisfechas, entre ellas la de un suministro de agua potable. Ante esta situación, la mayoría de las viviendas se abastecen de agua de forma mixta, es decir, de los jagüeyes y de fuentes alternas como agua lluvia, agua transportada en carrotanques e incluso de pimpinas de agua transportada desde la zona urbana de Sincelejo. Estas prácticas sumadas a los hábitos de consumir el agua cruda, usar el agua del jagüey para el lavado de frutos de pancoger y usar el agua del jagüey para el lavado de los utensilios de cocina, podrían aumentar el riesgo de contraer toxoplasmosis, lo que comprometería la salud de neonatos de madres primoinfectadas y de personas inmunodeprimidas habitantes de la zona de estudio.

En cuanto a la presencia de gatos, a pesar de que no se encontró una asociación estadística con la frecuencia de detección de *T. gondii,* el hecho de que en la finca del corregimiento de Cruz del Beque en donde había ocho gatos como mascotas fuera la única con muestras positivas para *T. gondii* en los dos tipos de muestra, evidencia el riesgo de contraer toxoplasmosis de las personas en este hogar. Sin embargo, no es posible afirmar que la contaminación del agua encontrada en este punto de muestreo se deba exclusivamente a la presencia de los gatos, pues no se hicieron estudios serológicos en estos animales.
